# Supplementary material for: Experiential Learning Interventions and Healthy Eating Outcomes in Children: A Systematic Literature Review
Source: Int J Environ Res Public Health. 2021 Oct 15;18(20):10824. doi: 10.3390/ijerph182010824 (PMC8535521; doi:10.3390/ijerph182010824)
Supplement: Supplementary file 1 [file ijerph-18-10824-s001.zip › Proofed Supplementary Table S2 .pdf]

Manuscript. Ref. No.: ijerph-1379741

Title: Experiential Learning Interventions and Healthy Eating Outcomes in Children: A Systematic Literature Review

Supplementary Table S2: Experiential learning strategies used by the included studies.

Activities	Types	Studies	*n*
Taste-testing	Fruits and/or Vegetables	[44], [52], [48], [39], [40], [45], [56], [47], [57]	9
	Healthy foods	[55]	1
	Snacks	[46]	1
	Foods	[59], [60], [56], [38], [43], [61], [62], [58]	8
Cooking	Preparing foods	[46], [53], [56]	3
	Fruit and vegetables	[45], [56]	2
	Snacks	[46]	1
	Other foods/meals	[52]	1
Calculations/recording	Sugar and fat	[55]	1
	Veggie math	[41]	1
	3-day fruit and vegetable intake	[56]	1
	Personal food pyramid	[38]	1
	Math activity	[54]	1
Shopping list and Food purchasing	Creating a shopping list	[55]	1
	Selecting food/meals	[56], [38], [43], [58]	4
	Simulated shopping	[38]	1
	Food classification	[38]	1
Sensory evaluation	Smell/touch/sight/sound of foods	[59], [60], [43], [54]	4
Gardening	Planting and harvesting	[52], [40]	2
Roleplay/modelling	Comic book	[46]	1
	Characters	[44]	1
	Role playing	[59], [47]	2
Games	Guessing food	[43], [39]	2
	Food labelling	[49]	1
	Competitions (eating fruits and vegetables)	[46]	1
	Card/Board games	[43], [51]	2
	Fun play	[56]	1
	Mystery bag	[54]	1
Songs		[59], [43], [50]	3
	Nursery rhyme	[60]	1
Storybook		[44], [46], [60], [54], [48]	5
	Making stories (characters)	[39]	1
Creative activities	Colouring	[59], [60]	2
	Drawing	[60], [54], [39]	3
	Collage	[39]	1
	Portraits	[39]	1
	Art and craft on F & V		
	Fruit and vegetable charts	[43]	1
	Posters/pamphlets	[51], [50]	2
Field trips	Imaginary trips/exploring places-eating fruits and vegetables	[59]	1
